# A New Insight into the Study of Neural Cell Adhesion Molecule (NCAM) Polysialylation Inhibition Incorporated the Molecular Docking Models into the NMR Spectroscopy of a Crucial Peptide–Ligand Interaction

**DOI:** 10.3390/biom16010019

**Published:** 2025-12-22

**Authors:** Ri-Bo Huang, Bo Lu, Si-Ming Liao, Xue-Hui Liu, Guo-Ping Zhou

**Affiliations:** 1 National Key Laboratory of Non-Food Biomass Energy Technology, Institute of Biological Science and Technology, Guangxi Academy of Sciences, 98 Daling Road, Nanning 530007, China; rbhuang@gxas.cn (R.-B.H.); lubo@gxas.cn (B.L.); simingliao@gxas.cn (S.-M.L.); 2 Rocky Mount Life Science Institute, Rocky Mount, NC 27804, USA; 3 Institute of Biophysics, Chinese Academy of Sciences, Beijing 100101, China; xhliu@ibp.ac.cn

**Keywords:** cancer cell migration, neuronal cell adhesion molecule (NCAM), cytidine monophosphate-sialic acid (CMP-Sia), polysialyltransferase (polyST), polysialyltransferase domain (PSTD), polybasic region (PBR), cytidine monophosphate (CMP), lactoferrin (LFcinB11), artificial intelligence (AI), Alphafold (AF), low molecular weight heparin (LMWH), molecular docking, NMR spectroscopy

## Abstract

The expression of polysialic acid (polySia) on the neuronal cell adhesion molecule (NCAM) is called NCAM-polysialylation, which is strongly related to the migration and invasion of tumor cells and aggressive clinical status. During the NCAM polysialylation process, polysialyltransferases (polySTs), such as polysialyltransferase IV (ST8SIA4) or polysialyltransferase II (ST8SIA2), can catalyze the addition of CMP-sialic acid (CMP-Sia) to the NCAM to form polysialic acid (polySia). In this study, the docking models of polysialyltransferase IV (ST8Sia4) protein and different ligands were predicted using Alphafold 3 and DiffDock servers, and the prediction accuracy was further verified using the NMR experimental spectra of the interactions between polysialyltransferase domain (PSTD), a crucial peptide domain in ST8Sia4, and a different ligand. This combination strategy provides new insights into a quick and effective screening for inhibitors of tumor cell migration.

## 1. Introduction

Polysialic acid (polySia) is a distinctive glycan, which is linked post-translationally to N-glycans on neural cell adhesion molecules (NCAMs) in mammalian cells [1,2,3,4,5,6,7,8,9]. These glycopolymer-containing glycoproteins are also expressed on human neuroblastoma cells [10,11,12].

It has been proposed that the polysialylation of NCAM needs to produce a sialic acid on NCAM through CMP sialic acid (CMP-Sia) catalysis, using a polysialyltransferase (polyST) [13,14,15,16,17,18,19,20,21,22,23,24,25], which is a suggested ‘new target’ for metastatic cancer [26]. As a transporter, polyST continuously carries more and more sialic acids to the NCAM to form polySia chains. This process is called NCAM polysialylation [23,27,28]. Thus, NCAM polysialylation may be related to the polyST–(CMP-Sia) interaction and polyST–polySia interaction in the lumen of the Golgi apparatus [29,30,31,32,33].

**Figure 1:**
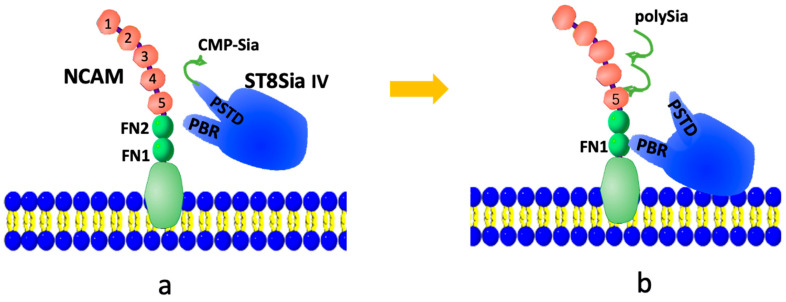
A possible modulation model of the NCAM polysialylation. At first, CMP-Sia is bound to the PSTD in ST8Sia IV (**a**), then sequentially transfers sialic acid (Sia) residues by polyST from multiple CMP-Sia molecules to synthesize the polySia chain, which is further released into NCAM (**b**).

The NCAM polysialylation pathway is illustrated in Figure 1. PolySTs (ST8Sia2 or ST8Sia4) initially bind to the activated donor substrate, CMP-Sia, via the polysialyltransferase domain (PSTD) (Figure 1a). The PSTD then catalyzes the synthesis of the polySia chain from CMP-Sia. Once the polySia chain is synthesized, the intramolecular interaction [33] between the polybasic domain (PBR) and PSTD within a polyST causes a conformational change in the PSTD, which leads to the release of the polySia chain into the NCAM from the PSTD (Figure 1b). 

ST8Sia II (STX) and ST8Sia4 (PST) are two highly homologous mammalian polySTs, which are members of the ST8Sia gene family of sialyltransferases [34,35,36,37,38,39,40]. Previous discoveries showed that both ST8Sia II (ST8Sia2) and ST8Sia IV (ST8Sia4) contain a polybasic motif of 32 amino acids, which is defined as the polysialyltransferase domain (PSTD) [41,42,43]. It has also been proposed that the PSTD is a crucial region within polysialyltransferase, involved in substrate binding and enzyme activity. The PSTD range in ST8Sia4 is from residue 261 to 292 in ST8Sia II, and from residue 246 to 277 in ST8Sia4, respectively. The evidence from in vitro studies strongly suggests that the PSTD plays a significant role for the active site and catalytic mechanism of polysialyltransferase (polyST) [34,37].

Chou’s Alpha-helical Wenxiang diagram [44,45,46,47,48,49,50,51,52,53] has been used to study protein–coil interactions [48,49], prediction of alpha-helical protein stability and the misfolding mechanism of the protein [48,49,50]. This may be helpful to further understand the molecular mechanism of NCAM polysialylation by incorporating the Wenxiang diagram into NMR spectroscopy, based on previous studies [50]. 

Considering the shorter non-helical domain in the PSTD, and the fact that the ligands interacting with the PSTD are usually small molecules, a combination method of an effective NMR experimental method and a molecular docking method is proposed. Cytidine monophosphate CMP), the low molecular weight heparin (LMWH), and LFconB11 have been proposed to be inhibitors of NCAM polysialylation. 

The previous NMR studies further supported that there is a specific interaction between an isolated PSTD peptide and CMP-Sia [50]. Recent studies have found that these two interactions could be inhibited by CMP, based on in vitro studies [54] and 1H-15N HSQC spectra [55,56], respectively.

Similarly, previous studies indicated that the LMWH binds strongly to an isolated PSTD peptide. It is suggested to be a more effective inhibitor than other forms of heparin, like the heparin tetrasaccharide (DP4). Overexpression of the NCAM polysialylation is linked to cancer cell migration, so inhibiting this process with the LMWH is a potential therapeutic approach. 

LFcinB11 is a synthetic, 11-residue peptide derived from the N-terminal residues of Lactoferrin, which can not only inhibit NCAM polysialylation through a PSTD-LFcinB11 interaction, but also can inhibit the formation of neutrophil extracellular traps (NETs): a network of extracellular strings of DNA that bind to pathogenic microbes [57,58,59,60,61,62,63,64,65,66,67,68,69,70,71]. Thus, LfcinB11 has been suggested to be a bifunctional inhibitor [57].

The determinations of the above inhibitors were mainly based on the interactions between these ligands and an individual PSTD peptide using NMR experiments. The question is whether the specificity of these interactions can be applied in the context of the entire protein molecule (ST8Sia4). In other words, if PSTD is not separated from the ST8Sia4 molecule, can these ligands still bind to the ST8Sia4 protein? If so, is the binding domain between ST8Sia4 and the three ligands mentioned above covered in the PSTD?

In order to answer this question, we would usually need to obtain the 3D structure of the ST8Sia4 protein, then determine whether these ligands such as CMP, LMWH, or LfcinB11 are specifically bound to the ST8Sia4. It has been known that both X-ray crystallography and NMR provide real-world protein–ligand structures. However, the 3D crystal or the NMR structures of ST8Sia2 and ST8Sia4 have not been determined, due to the presence of the large numbers of hydrophobic amino acid residues in the membrane environment and random domains in these polySTs, which make it difficult to obtain qualified experimental samples for the X-ray crystallography and NMR experiments [72].

Mankind has entered an era of rapid development of artificial intelligence (AI), which can accurately predict the 3D structure of any protein–ligand docking models, and screen ideal drugs or inhibitors in a short time, dramatically accelerating the drug discovery process. It should be pointed out that the accuracy of predicting protein structures and protein–ligand docking models using AI software is still limited by factors such as protein flexibility, the need for large, diverse training datasets, and the challenge of generalizing to novel proteins and ligands that are not seen during training. Thus, the accuracy of the prediction needs to be further verified.

Computational pharmaceutics is playing a rapidly growing role in accelerating drug discovery and development, enhancing efficiency, and enabling personalized medicine. However, it faces limitations related to data quality, the complexity of biological systems, and the need for experimental validation [73,74,75]. 

Recent studies indicate that NMR is a powerful tool in drug discovery for studying peptide–ligand interactions, as it can provide atomic-level detail on structure, dynamics, and binding modes, even for weak interactions [76,77,78]. The key applications of NMR in drug discovery are as follows: identify and characterize fragments that weakly bind to a target protein, which is crucial for fragment-based drug design (FBDS); provide information on the 3D structure of the protein–ligand complex, its dynamics, and conformational changes upon binding; and guide the optimization of drug leads by providing structural feedback. In addition, combining NMR with techniques like cryo-EM or X-ray crystallography can overcome the limitations of each individual method, particularly for high-molecular-weight proteins and complexes. However, the size of the protein target has been a major limitation for NMR, although recent technical advances have extended the mass limit.

In order to verify the accuracy or reliability of the predicted ST8Sia4–ligand docking model in a shorter time, and to speed up the search for promising drug candidates, a new and faster approach for analyzing the accuracy of docking models is proposed in this study. The approach is a combination of the predicted molecular docking models of ST8Sia4–ligands by AI and chemical shift perturbations of the PSTD–ligand interactions by a short-time NMR experiment. As a target peptide, the use of PSTD for its interaction with the ligands can overcome the limitations of the large ST8Sia4 size and the time-consuming NMR. 

The results displayed that the docking models between these ligands and the ST8Sia4 protein are completely consistent with the NMR experimental results of the interactions between an individual PSTD peptide and these ligands. These analyzing results suggest that the NMR data of the interaction between an active site domain peptide and its ligand may be used to verify the accuracy of the predicted molecular model between the whole protein and the inhibitor. Furthermore, the binding affinity between the ST8Sia4 molecule and these ligands could be indirectly determined, and the more powerful inhibitor could also be screened out.

## 2. Materials and Methods

### 2.1. The Prediction of 3D Target Molecule (ST8Sia4) Model

The 3D ST8Sia4 model was predicted using Alphafold 2 (v2.3.0), an artificial intelligence system developed by DeepMind [79,80]. The amino acid sequence of the ST8Sia4 protein was inputted into the AlphaFold server to produce its three-dimensional structure. AlphaFold uses deep learning and coevolutionary data from multiple sequence alignments of related proteins to calculate the 3D coordinates of the protein’s atoms, providing researchers with a detailed model of the protein’s native state and its potential function [80].

### 2.2. The Prediction and Visualization of 3D ST8Sia4–Ligand Docking Models 

The docking models of ST8sia4 and two ligands (CMP, LMWH) were predicted using the DiffDock 2.1.0 [81]. DiffDock is more accurate for protein–small molecule docking because it is specifically designed for that task, using a diffusion-based model to generate poses and an energy model for scoring. The above ST8Sia4 PDB file from Alphafold2 (AF2) and the chemical structures of the ligands (e.g., in SDF format) were input into this server to calculate the docking structures of ST8Sia4 and the ligands, using the Neurosnap platform. The docking model of the ST8sia4–LFcinB11 peptide was obtained using Alphafold3 [82,83,84,85,86,87]. AlphaFold 3 (AF3) predicts protein–peptide docking with higher accuracy than DiffDock, due to a fundamentally different approach to the problem. While DiffDock treats docking as a geometric sampling and refinement task, AF3 uses a unified, all-in-one generative model that co-folds the protein and peptide together. This allows AF3 to account for the structural flexibility of both molecules during binding: a major challenge for traditional docking methods. The sequences of ST8Sia4 and LFcinB11 were input into this server. The predicted 3D structures of all complexes were visualized using the PyMol 3.1 server (https://www.pymol.org). 

Compared with AF3, other docking software such as AutoDock Vina cannot fully predict protein flexibility. Because AutoDock Vina treats the protein as a rigid structure, though it can handle flexibility in selected side chains [88,89,90,91,92,93,94,95]. For systems with larger protein motions, alternative methods like ensemble docking (which involves docking against multiple protein conformations) or simulations using molecular dynamics are necessary. 

### 2.3. The Indirect Measurement of the Binding Affinity Between ST8Sia4 and the Different Ligands

PyMOL can also be used to perform qualitative and semi-quantitative analyses on protein–ligand docking models, and it provides indirect evidence for the binding affinity between the protein and the ligand. By visually inspecting the complex, measuring distances between protein and ligand atoms, and analyzing potential non-covalent interactions, the binding strength between ST8Sia4 and each ligand can be inferred. The primary distance range for the strongest hydrophobic interactions between a protein and a small ligand is 3.0 to 4.5 Å, which corresponds to favorable van der Waals contact. This distance range can be extended to 5.1 Å for the hydrophobic interaction with the medium-range category. Usually, hydrogen bonds are generally 3.5 Å or less. 

### 2.4. Chemical Shift Perturbations (CSPs) of NMR Spectra

CSPs of NMR were used for analyzing biomolecular interactions such as those between protein and ligands. CSPs pinpoint the specific binding sites of the interactional molecules by measuring the chemical shift change in nuclei in a 15N-labeled protein or enzyme [96,97,98]. This technique is crucial for understanding the dynamics of biomolecular complexes in various biological processes. In previous studies, the 1H-15N HSQC and 1H-13C HSQC spectra of the PSTD–(CMP-Sia), the PTSD–CMP, the PTSD–LMWH, and the PTSD–Lactoferrin (LFcinB11) interactions have been obtained [38,52,53]. In this study, two sets overlaid the 1H-15N HSQC spectra of the PSTD in the absence of any ligands, and the presence of CMP, LMWH, or LfcinB11 were, respectively, displayed to determine which ligand was likely to be the strongest inhibitor of NCAM polysialylation.

## 3. Results

### 3.1. The Visualization and Confidence of the Predicted 3D ST8Sia4 Model

**Figure 2:**
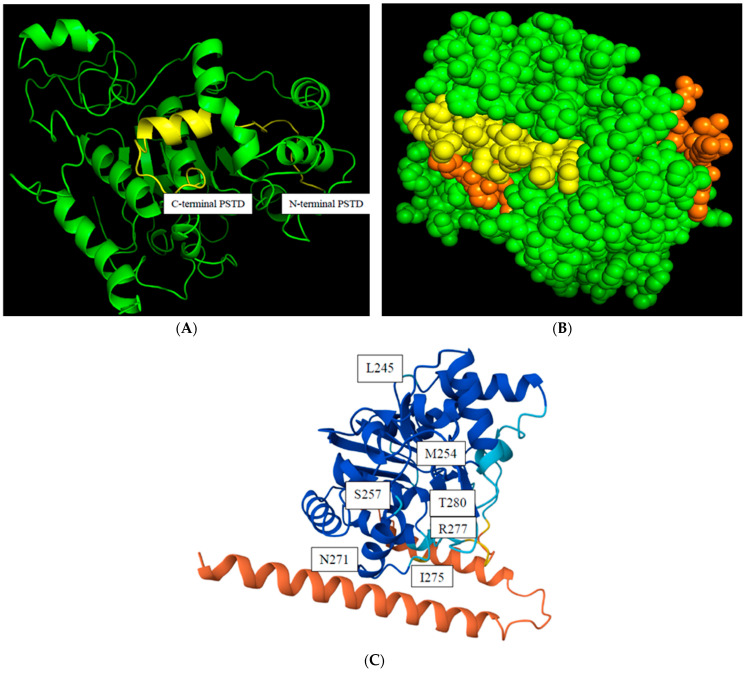
The predicted model of a 3D ST8Sia4 structure. The backbone structure of the. ST8Sia4 (green), and the whole PSTD within ST8Sia4 is marked in yellow (**A**); the surface sphere model of the ST8sia4, in which the N-terminal and C-terminal regions of the PSTD were marked in orange, and the middle region of the PSTD are marked in yellow (**B**); and the color-coded confidences of the 3D ST8Sia4 model displayed the highest confidence domain (blue), the lowest confidence domain (orange), and the middle confidence domain (light blue) for the ST8Sia4 structure (**C**).

The predicted 3D ST8Sia4 model is generated using Alphafold and was visualized in the PyMol 3.1 window. The PSTD (246K-277R) is labeled in yellow (Figure 2A), and the surface sphere model of this 3D structure further shows that the whole PSTD is basically exposed on the outermost side of the entire ST8Sia4 molecule, particularly for the N-terminal residue range of the PSTD (Figure 2B). This surface feature may facilitate the direct binding of proteins and ligands.

The predicted local distance difference test (pLDDT) score is a measure of confidence in a predicted protein structure model generated by AlphaFold. The pLDDT score ranges from 0 to 100, with higher values indicating greater confidence that the predicted coordinates for an amino acid residue agree with the actual protein structure. As shown in Figure 2C, the N-terminal ST8Sia4 is displayed as red, indicating the lowest confidence (pLDDT < 50) in the protein due to its highly flexible and unstable structural region. In comparison, most dark blue regions, including almost the whole PSTD, displayed a very high level of confidence (pLDDT > 90). Thus, the predicted ST8Sia4 model is basically suitable to be used for predictions of the protein–ligand docking models, due to the highest confidence being in the whole ST8Sia4 core, excluding its N- and C-terminal domains.

### 3.2. The Predicted Docking Models of the Three Ligands Bound to the ST8Sia4 Molecule

**Figure 3:**
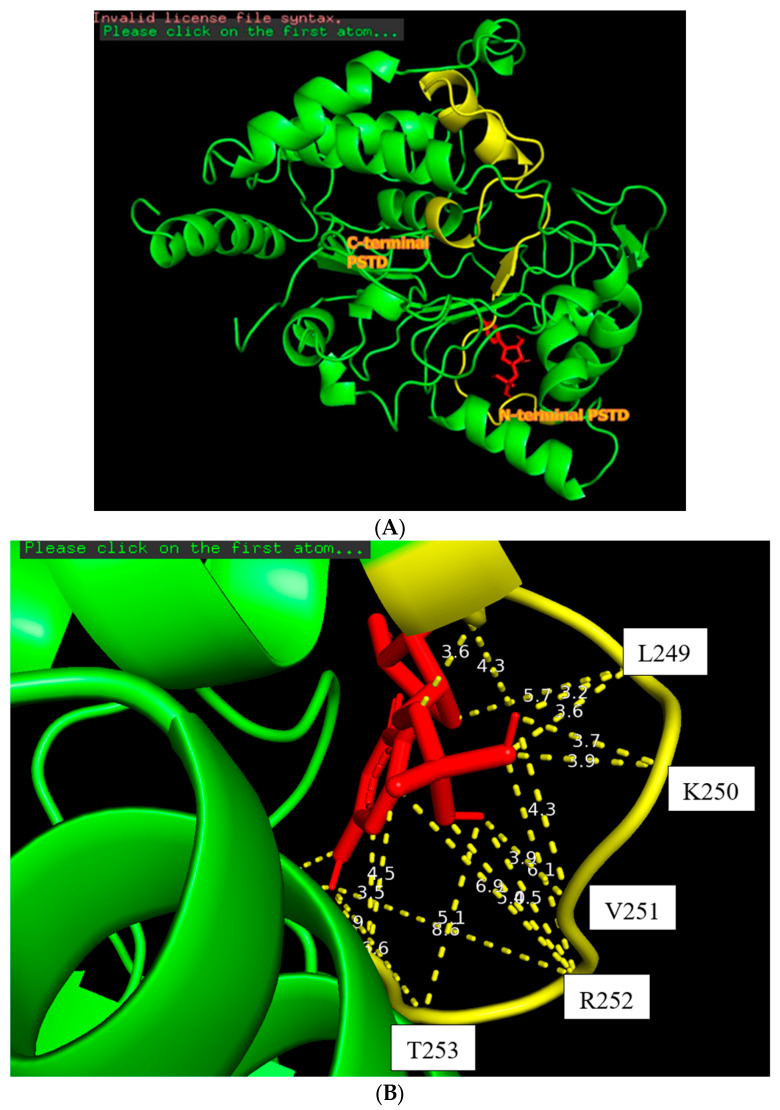
The docking of the CMP molecule (red) bound to the N-terminal PSTD (yellow) in the ST8Sia4 molecule (**A**); the displayed distances between CMP and the binding sites of the N-terminal PSTD (**B**).

**Figure 4:**
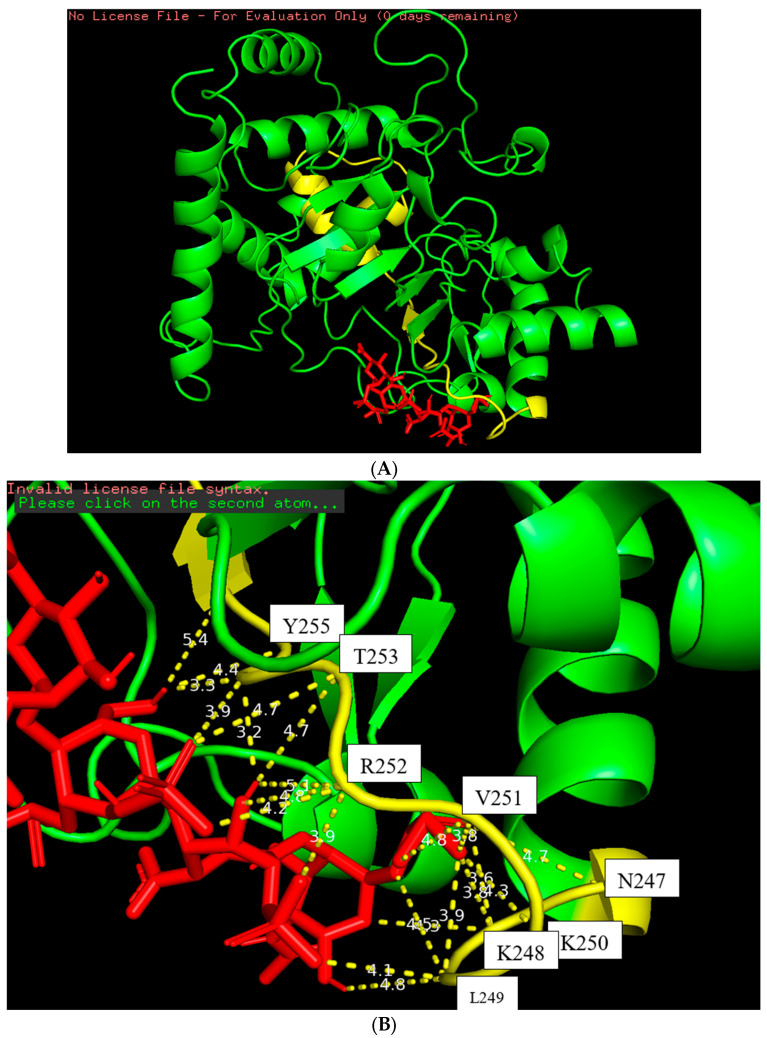
The docking figure of the LMWH molecule (red) bound to the N-terminal PSTD (yellow) in the ST8Sia4 molecule (**A**); the displayed distances between LMWH and the binding sites of the N-terminal PSTD (**B**).

**Figure 5:**
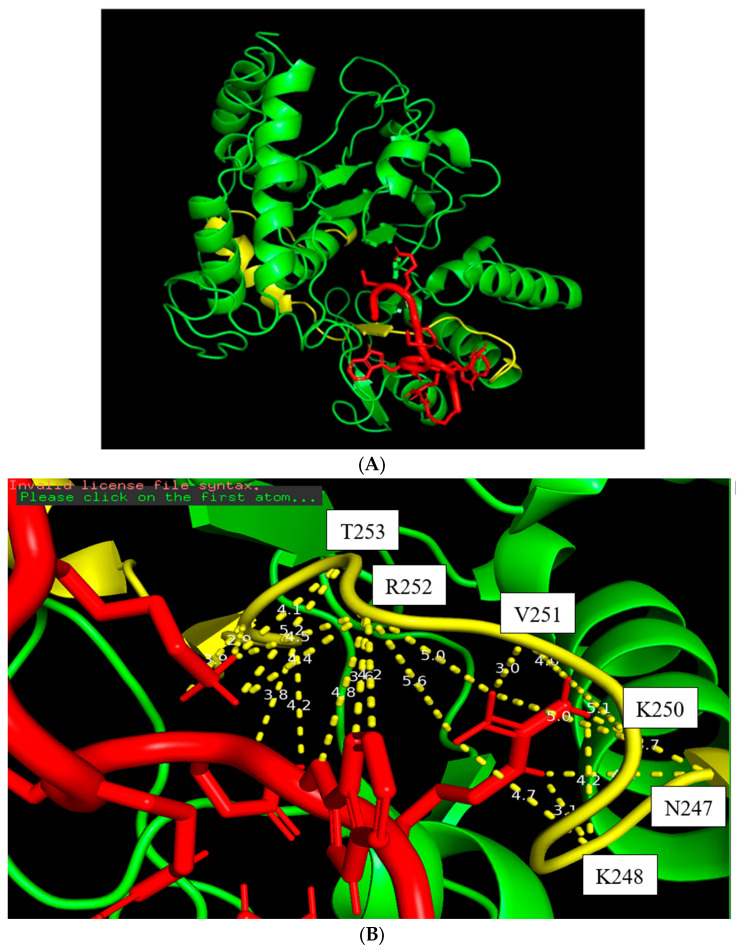
The docking figure of the LfcinB11 molecule (red) bound to the N-terminal PSTD (yellow) in the ST8Sia4 molecule (**A**); the displayed distances between LfcinB11 and the binding sites of the N-terminal PSTD (**B**).

There are three main interaction forces between ST8Sia4 and the three ligands, CMP, LMWH, and LFcinB11, according to the predicted docking models of ST8Sia4–CMP, ST8Sia4–LMWH, and ST8Sia4–LfcinB11 (Figure 3, Figure 4 and Figure 5). They are as follows: the moderate hydrogen bond interaction (2.5–3.5 Å), a weak hydrogen bond/van der Waals interaction (with the optimal attraction) occurring in the range (3.5–4.0 Å), and hydrophobic interaction (4.0–6.9 Å).

#### 3.2.1. The Moderate Hydrogen Bond Interaction Between Three Ligands and the PSTD of ST8Sia4 Molecule

The docking model of ST8Sia4–CMP is shown in Figure 3. The whole CMP molecule (red) was bound to the N-terminal region of the PSTD (Figure 3A). L249 and CMP can form an H-bond because the amino acid Leucine has a hydrogen bond (the N-H in the backbone or side chain), and CMP has hydrogen bond acceptors (oxygen and nitrogen atoms in its phosphate and base, respectively). Similarly, A254 has a polar backbone that can participate in a hydrogen bond formation with the polar group in the ribose and phosphate of the CMP molecules. As shown in Figure 3B, there are only two moderate hydrogen bond interactions detected, between CMP and L249 and CMP and A254. The interaction distances of these two pairs are 3.2 and 2.9 Å, respectively (Table 1).

The docking model of ST8Sia4–LMWH is shown in Figure 4. The whole LMWH molecule (red) was bound to the N-terminal region of the PSTD (Figure 4A). As shown in Figure 4B, there is only one moderate hydrogen bond interaction that was detected between LMWH and A254 of the PSRD in the ST8Sia4 protein (Table 1).

The docking model of the ST8Sia4–LFcinB11 peptide is shown in Figure 5. The whole LFcinB11 peptide molecule (red) was also bound to the N-terminal region of the PSTD (Figure 5A). As shown in Figure 5B, there are three moderate hydrogen bond interactions that were detected between LFcinB11 and K248, LFcinB11 and V251, and LFcinB11 and A254. The interaction distances of these three pairs are 3.1, 3.0, and 2.9 Å, respectively (Table 1).

#### 3.2.2. The Weak Hydrogen Bond/Van der Waals Interactions Between Three Ligands and the PSTD of ST8Sia4 Molecule (3.5–4.0 Å)

As shown in Figure 3B, four weak hydrogen bond interactions were detected between CMP and the residues K248, L249, K250, and V251. The interaction distances of these four pairs are 3.6, 3.9, 3.9, and 3.9 Å, respectively (Table 1).

Six weak hydrogen bond interactions were detected between LMWH and the residues K248, L249, V251, R252, and A254, in which two weak hydrogen bond interactions were detected between LMWH and K248 (3.6 and 3.8 Å), and the contributions of other four weak hydrogen bond interactions come from the residues L249, V251, R252, and A254, and their interaction distances are 3.9, 3.8, 3.9 and 3.9 Å, respectively (Figure 4B and Table 1).

Five weak hydrogen bond interactions were detected between LFcinB11 and residues N247, V251, R252, A254, and Y255, and the distances of these five pair interactions are 3.7, 4.0, 3.6, 3.9, and 3.8 Å (Figure 5B and Table 1).

#### 3.2.3. The Hydrophobic Interactions Between Three Ligands and the PSTD of ST8Sia4 Molecule (4.0–6.9 Å)

There are 11 hydrophobic interactions between CMP and seven residues in the PSTD: N247, K248, V251, R252, T253, A254, and Y255 (Figure 3B and Table 1). Particularly, R252 contributed four hydrophobic interactions, which is more than the other residues for CMP–ST8Sia4 docking.

There are 14 hydrophobic interactions between LMWH and eight residues in the PSTD: N247, K248, L249, K250, V251, R252, T253, and Y255 (Figure 4B and Table 1). Particularly, R252 also contributed four hydrophobic interactions, which is more hydrophobic interactions than others for LMWH–ST8Sia4 docking.

There are 15 hydrophobic interactions between LFcinB11 and seven residues in the PSTD: N247, K248, L249, K250, R252, T253, and Y255. Similarly, R252 contributed six hydrophobic interactions, which was also more hydrophobic interactions than others for LFcinB11–ST8Sia4 docking (Figure 5B and Table 1). 

### 3.3. The Main Features of Chemical Shift Perturbation (CSP) Distribution in the PSTD upon Ligand Binding

The CSP distribution graphic based on the NMR experiments is a visual representation that shows the magnitude of chemical shift changes for each residue in a biomolecule (like a protein) after it interacts with another molecule, such as a ligand or another protein. This distribution highlights the specific regions of the molecule that are involved in the interaction, suggesting valuable information for binding sites, mapping conformational changes, and binding affinities by observing which residues undergo the most significant shifts.

**Figure 6:**
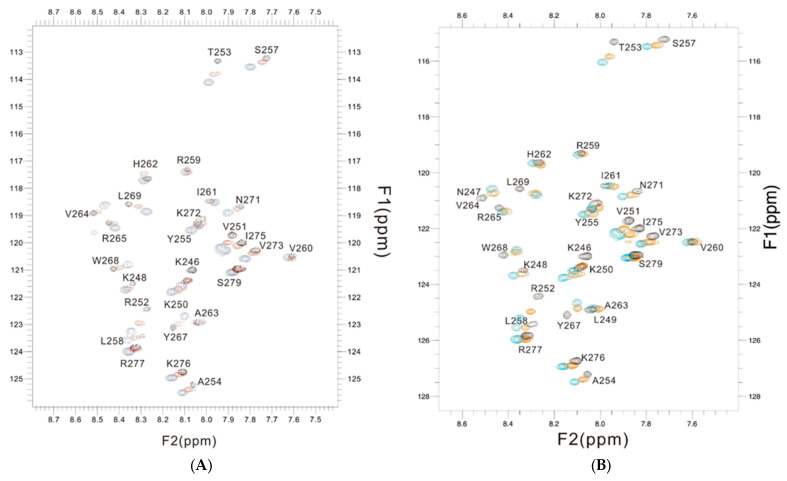
The overlaid 1H-15N HSQC spectra of the 2 mM PSTD (black) in the absence and presence of 1mM CMP (red) and 80 μM LFcinB11 (blue) (**A**); the overlaid 1H-15N HSQC spectra of the 2 mM PSTD in the absence (black) and presence of 80 μM LMWH (red) and 80 μM LFcinB11 (blue) (**B**), respectively.

The changes in chemical shift in the PSTD for the PSTD–CMP and PSTD–LMWH interactions, and that of the PSTD, PSTD–LMWH, and PSTD–LfcinB11 interactions, are shown in the overlaid 1H-15N HSQC spectra Figure 6A,B, which qualitatively displayed that the chemical shift values of the PSTD–LfcinB11 interaction were larger than that of the PSTD–CMP and the PSTD–LMWH interactions. 

**Figure 7:**
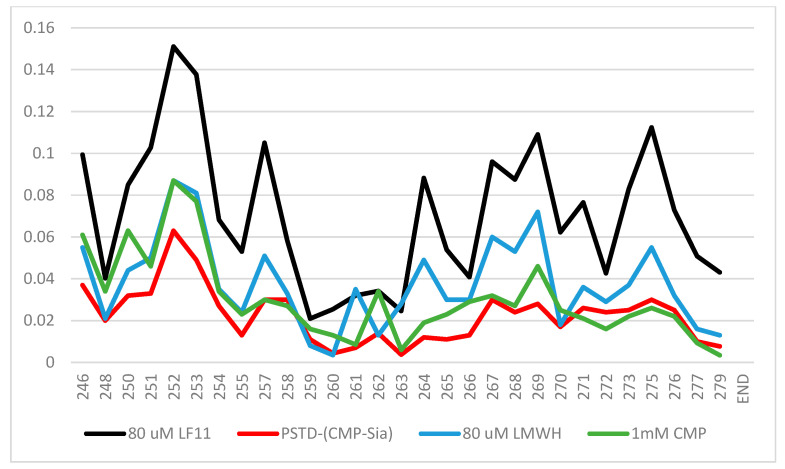
The chemical shift perturbation (CSP) curves of the PSTD–1 mM (CMP-Sia), the PSTD–1 mM CMP [52], the PSTD–80 μM LMWH, and the PSTD–60 µM LfcinB11 interactions were obtained according to the previous NMR experiments [35,38,53], and they are combined in a single figure.

According to the previous 1H-15N HSQC spectra [38,52,53], the CSPs of the PSTD–1mM (CMP-Sia), the PSTD–1mM CMP, and 60 μM LfcinB11 interactions were further quantitatively shown in Figure 7, in which the range of the largest CSP values is from N247 to Y255 at the N-terminus. Secondly, the largest CSP values for these three interactions are displayed in residue R252 (Figure 7). 

Obviously, the CSPs for the PSTD–(CMP-Sia) interaction are the smallest compared with those of the interactions between the PSTD and CMP and LMWH and LfcinB11, respectively. These results suggest that these inhibitors for the inhibition of NCAM polysialylation, in order from the strongest to weakest, are LfcinB11, LMWH, and CMP. 

## 4. Discussion

### 4.1. The Rationale of Choosing a Predictive 3D ST8Sia4 Model as a Template for the Docking Models Between Itself and the Ligands

It has been proposed that the predicted 3D ST8Sia4 structure is quite accurate, using the ST8Sia3 crystal structure (code: c5bo6B) as a template [32]. The updated 3D structure model of ST8Sia4 (Figure 2), using the Alphafold (AF) server, is more accurate than the earlier model based on the confidence analysis. Therefore, it is reasonable that the predicted ST8Sia4 pdb file was preferentially selected as a structural template to predict the ST8Sia4–ligand docking models. In addition, the PSTD is significantly exposed on the outer surface of the 3D ST8Sia4 structure (Figure 2B,C), which facilitates ligand accessibility. For extracellular receptors or enzymes in the cytoplasm, this means the binding site must be on the solvent-exposed surface for an efficient interaction of the protein–ligand.

### 4.2. The Main Features of the Predicted ST8Sia4–Ligand Docking Models

The docking models from Figure 3, Figure 4 and Figure 5 clearly displayed that all ligands (CMP, LMWH, and LfcinB11) were specifically bound to the PSTD region of the ST8Sia4 molecule. These results further supported the previous discovery that PSTD is the binding domain of the polyST [38,52,53], and all three ligands, CMP, LMWH, and LFcinB11, are all bound to the residual range from N247 to Y255 (Table 1). As shown in Table 1, there are 17 binding sites for ST8Sia4–CMP docking and 20 binding sites for ST8Sia4–LMWH docking, respectively. The most binding sites are shown in the ST8Sia4–LFcinB11 docking model, which has 24 binding sites (Table 1). According to Figure 2B, Figure 3B and Figure 4B, the average binding distances between ST8Sia4 and CMP, LMWH, and LFcinB11 are 4.55, 4.33, and 4.23 Å, respectively (Table 1). The shortest average distance indicates **a** tighter, more stable, and more complementary fit between the ST8Sia4 and LFcinB11, which suggests strong, favorable interactions that hold the ligand LFcinB11 securely in the ST8Sia4 protein binding pocket. In contrast, the longest distance for ST8Sia4–CMP reflects a looser and less complementary fit. 

Particularly, residue R252 contributed more interaction compared with other residues in the N247–Y255 range (Table 1). For example, R252 contributed four hydrophobic bonds for ST8Sia4–CMP docking, and L249 only contributed one hydrogen bond (3.2 Å) and one hydrophobic or weak hydrogen bond (3.9 Å) interaction for ST8Sia4–CMP docking. R252 contributed five hydrophobic interactions and one weak hydrogen bond for ST8Sia4–LMWH docking, which is also more than the other residual contributions for the same docking interaction. Moreover, R252 contributed six hydrophobic interactions and one hydrogen bond for ST8Sia4–LFcinB11 docking, which is much more than the contributions of any other residues.

In essence, the R252 side chain’s flexibility and nonpolar patches allow it to act as a versatile anchor that can bridge both polar and non-polar regions, thus contributing to several hydrophobic interactions with a docked ligand. This multimodal character makes Arginine a frequent “hot spot” residue in protein–ligand binding interfaces. 

These results suggest that R252 is located near the binding site and has experienced a significant change in its local electronic environment upon the ligand binding, due to more non-covalent interactions. So many hydrophobic interactions formed on the R252 side chain may be a major driving force for binding strength and involve the burial of nonpolar surfaces, which changes the solvent exposure and, thus, the chemical shift.

Although a hydrophobic interaction is weaker than an electrostatic interaction, multiple weak interactions can collectively result in a strong and highly specific binding affinity and likely cover a larger surface area. Their cumulative effect could easily match or exceed the total energy of one strong and one weak electrostatic interaction in a biological context. According to this analysis, the residue R252 should experience a significant chemical shift perturbation (CSP) and a change in dynamics. It can also be associated with a conformational change in the protein as a whole or the local loop it is situated in. This speculation could be verified by the NMR experimental results.

### 4.3. Consistency Between the Predicted ST8Sia4–Ligand Models and NMR Experimental Results of PSTD-Ligand Interactions

It is known that the PSTD is critical for polyST (ST8Sia2 or ST8Sia4) enzymatic activity, and specifically for the elongation of polysialic acid (polySia) chains. Recent studies have confirmed that specific amino acid residues within the PSTD interact directly with both CMP-Sia and the growing polySia chain [35]. Therefore, it is reasonable to evaluate the interaction between the whole polyST molecule and its ligands through analysis of the NMR chemical shift perturbations (CSPs). 

After comparing the molecular docking models of ST8Sia4 and the three ligands, the binding site ranges of CMP, LMWH, and LFcinB11 on the ST8Sia4 are not only the same (N247–Y255) but are also almost consistent with the displayed range of the largest CSPs (N247–Y255) by the NMR experiments (Table 2). 

As shown in Figure 7, the largest CSP ranges are all from residues N247 to Y255, according to the NMR experimental results for the ST8Sia4–CMP, ST8Sia4–LMWH, and ST8Sia4–LFcinB11 interactions, which are also exactly the binding site residue range of the predicted docking models for these three ligands and the ST8Sia4 protein (Table 1).

The CSPs values for the PSTD are the smallest for the PSTD–(CMP-Sia) interaction [35]; thus, the PSTD–(CMP-Sia) interaction should be inhibited by CMP, LMWH, and LfcinB11. 

Larger CSPs are often associated with stronger, saturable binding (lower $K_{d}$) when the interaction is in the fast exchange regime on the NMR timescale. If both interactions are in fast exchange, the one with larger observed CSP values has a lower *K_d_* (higher affinity). Considering that very low concentrations of LMWH (80 uM) and LFcinB11 (80 uM) were used to interact with the PSTD peptide, and the CSP values of both the ST8Sia4–LMWH and ST8Sia4–LFcinB11 interactions are larger than that of the ST8Sia4–CMP (1mM) interaction, this indirectly suggests that the smallest *K_d_* value (high affinity) should be for ST8Sia4–LFcinB11 binding and the largest *K_d_* value should be for the ST8Sia4–CMP interaction.

Likewise, for the predicted docking models, the highest number of binding sites and the shortest average distance between the ligand and its binding sites on the protein suggest the best fit and the strongest intermolecular forces between ST8Sia4 and LFcinB11, and the lowest number of binding sites and the longest average distance reflect the weakest intermolecular forces between ST8Sia4 and CMP (Table 1).

Furthermore, the largest CSP peak value for these three ST8Sia4–ligand interactions are all displayed by residue R252 (Figure 7), which corresponds in accordance with the prediction’s docking models (Figure 3, Figure 4 and Figure 5). Thus, R252 is further confirmed to be at the direct binding interface of the predicted docking models, or the most significant conformational changes occurred in this residue and its associated region.

The consistency between the predicted ST8Sia4–ligand models and NMR experimental results of PSTD-ligand interactions is summarized in Table 2.

It is also known that the predictions for the binding affinity of most molecular docking models are not accurate, due to the limitations in the scoring function for calculations of affinity [69,70,71,72]. Scoring functions used in molecular docking often fail to simulate complex protein–ligand interactions fully and accurately, leading to biases and inaccuracies in virtual screening and target predictions [71]. On the other hand, although measuring the shortest distance between ligand and protein atoms is a component of predicting the binding affinity, it is not sufficient on its own. This is because it is only a single geometric measurement, whereas protein–ligand binding is a complex, dynamic process, driven by a variety of chemical and physical forces.

Chemical shift perturbation (CSP) of NMR spectroscopy is a dynamic measurement technique used to study protein–ligand interactions by monitoring how a ligand’s binding alters the NMR chemical shifts in the protein. This method relies on comparing NMR spectra of a protein or peptide with and without the added ligand, where significant changes in peak positions (chemical shift perturbations) indicate a binding site or a conformational change. Thus, combining CSP data and the affinity distance measurement of protein–ligand can provide a better prediction accuracy for protein–ligand docking models. 

Both molecular dynamics simulations (MDS) and NMR experiments are two efficient approaches to detect protein–ligand interactions. MD simulations provide atomistic details of the protein–ligand complex over time, offering insights into the dynamics and transient intermediates that are often difficult to capture solely through experimental methods [69]. NMR, in turn, provides highly accurate atomic resolution data on protein–ligand interactions and dynamics. The accuracy of the MD method could be validated with experimental data, such as that from the CSPs of NMR experiments. Therefore, by integrating these two powerful techniques, a more comprehensive and accurate understanding of the complex molecular mechanisms governing polyST–ligand interactions can be obtained in further studies.

## 5. Conclusions

In the predicted ST8sia4–CMP, ST8Sia4–LMWH, and ST8sia4–LFcinB11 docking models, the binding regions in the ST8Sia4 protein, including the PSTD, were accurately predicted, and this was supported by the NMR experimental results. The high consistence for the predicted docking models and the CSP analysis of the NMR experiments significantly strengthens confidence in the proposed binding interface and the overall binding model. These findings provide new insights into quick and effective screening for inhibitors of tumor cell migration.

## Figures and Tables

**Table 1:** The main features of the predicted ST8Sia4–ligand docking models and the predicted local distance difference test (pLDDT) scores. In this table, R252 (labeled by bold) forms the most binding sites with the ligand.

The Ligand Bound to the PSTD in ST8Sa4	Residue Range Bound to the Ligand	The Residue Name Contributed to Moderate H-Bonds and Interaction Distance (2.7–3.3 Å)	The Residue Name Contributed to Weak H-Bond/van der Waals Interaction and Interaction Distance (3.3–4.0 Å)	The Residue Name Contributed to the Hydrophobic Interaction and Distance (4–10 Å)	The Total Binding Sites of the Ligand on ST8Sia4	The Average Distance Between Each Ligand and the Binding Sites on ST8Sia4 (Å)	The Highest Level of Confidence for Predicted ST8Sia4–Ligand Docking Models
CMP 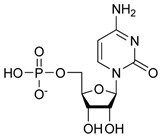	N247–Y255	L249: 3.2 Å; A254: 2.9 Å	K248: 3.6 Å L249: 3.9 Å K250: 3.9 Å V251: 3.9 Å	N247: 4.3, 5.0 Å; K248: 5.1 Å; V251: 4.3 Å; **R252: 4.5, 5.0, 6.1, 6.9 Å;** T253: 5.1 Å; A254: 4.9 Å: Y255: 4.8 Å	17	4.55	pLDDT > 90
LMWH 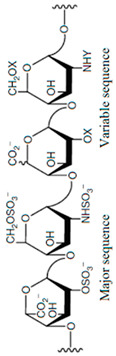	N247–Y255	R252: 2.4 Å A254: 3.3 Å	K248: 3.6, 3.8 Å; L249: 3.9 Å; V251: 3.8 Å; R252: 3.9 Å;	N247: 4.7 Å: K248: 4.3 Å; L249: 4.1, 4.5, 4.8 Å; K250: 4.3 Å; V251: 4.8 Å; **R252: 4.2, 4.8, 4.8, 5.1, 6.0 Å;** T253: 4.7, 4.7 Å; Y255: 4.4 Å	21	4.33	pLDDT > 90
LFcinB11 peptide sequence: RRWQWRMKKLG	N247–Y255	K248: 3.1 Å V251: 3.0 Å A254: 2.9 Å	N247: 3.7 Å V251: 4.0 Å R252: 3.6 Å A254: 3.9 Å; Y255: 3.8 Å	N247: 4.6 Å K248: 4.2, 4.7 Å L249: 5.1 Å K250: 5.0, 5.1 Å **R252: 4.2, 4.4, 4.5, 4.8, 5.0, 5.6 Å;** T253: 4.1, 5.2 Å; Y255: 4.2 Å	23	4.23	pLDDT > 90

**Table 2:** The comparison of main features between predicted ST8Sia4–ligand models and experimental NMR results of PSTD-ligand interactions.

The Ligand Name Bound to the PSTD in ST8Sia4	The Binding Range of the Predicted ST8Sia4–Ligand Docking (Figure 3, Figure 4 and Figure 5)	The Range of the Largest CSPs, Determined by the NMR Experiments (Figure 7)	The Residue Displayed Largest CSP Peak and the Most Binding Sites (Figure 3, Figure 4 and Figure 5)	The Residue Name and the Largest CSP by NMR Experiment (Figure 7)
CMP	N247–Y255	N247–Y255	R252: 4 binding sites	R252: 0.087
LMWH	N247–Y255	N247–Y255	R252: 6 binding sites	R252: 0.087
LFcinB11	N247–Y255	N247–Y255	R252: 7 binding sites	R252: 0.151

## Data Availability

The original contributions presented in this study are included in the article. Further inquiries can be directed to the corresponding author.

## Abbreviations

polySia	polysialic acid
Sia	mono sialic acid
CMP-Sia	cytidine monophosphate-sialic acid
CMP	cytidine monophosphate
NCAMs	neural cell adhesion molecules
polySTs	polysialyltransferases (ST8Sia II (STX) and ST8Sia4 (PST)
PSTD	polysialyltransferase domain
CSP	chemical shift perturbation
